# Predictors of immunotherapy benefit in Merkel cell carcinoma

**DOI:** 10.18632/oncotarget.27823

**Published:** 2020-11-24

**Authors:** Alec J. Kacew, Harita Dharaneeswaran, Gabriel J. Starrett, Manisha Thakuria, Nicole R. LeBoeuf, Ann W. Silk, James A. DeCaprio, Glenn J. Hanna

**Affiliations:** ^1^GJS Associated with Laboratory of Cellular Oncology, CCR/NCI, Bethesda, MD, USA; ^2^Dana-Farber Cancer Institute, Boston, MA, USA; ^*^These authors contributed equally to this work

**Keywords:** cancer, genomics, immunotherapy, Merkel cell carcinoma, precision medicine

## Abstract

Merkel cell carcinoma is a rare cancer for which immune checkpoint blockade is standard-of-care for recurrent/metastatic disease. However, not all patients benefit from immunotherapy. A greater understanding of molecular mechanisms and predictive biomarkers are unmet needs. We retrospectively analyzed electronic health records and next-generation sequencing data of 45 patients treated at our institution from 2013 to 2020 to understand clinical and genomic correlates of benefit from immunotherapy. Our cohort predominantly included individuals with stage III disease at primary disease diagnosis and individuals with stage IV disease at recurrent/metastatic disease diagnosis. Most received immunotherapy as first-line treatment. 43% experienced objective response (median duration of response 24.2 months, 95% confidence interval 8.8-not reached). Median overall survival was 15.5 months (95% confidence interval 9.0–28.7) (median follow-up 25.2 months). Less advanced stage at primary disease diagnosis and shorter disease-free interval between completion of initial treatment and recurrence were each associated with greater odds of response (odds ratio of 0.06, *p* = 0.04 for stage; odds ratio 0.75, *p* = 0.05 for disease-free interval). Single-nucleotide variants in *ARID2* and *NTRK1* were associated with response (*p* = 0.05, without Bonferroni correction), while none of Merkel cell polyomavirus status, total mutational burden, ultraviolet mutational signatures, and copy-number alterations predicted outcomes. Patients with shorter disease-free interval may be particularly suitable immunotherapy candidates. Our molecular findings point to *ARID2* and *NTRK1* as potential predictive markers and/or therapeutic targets (e.g., with Trk inhibitors), although this association needs to be confirmed in a larger sample.

## INTRODUCTION

Merkel cell carcinoma (MCC) is a relatively rare cancer, with roughly 400 cases per 100,000 persons each year in the United States [1]. Before the age of immunotherapy, there was significant room for improvement in survival rates, with five-year survival of 60% overall and 14–21% among patients with distant disease [2, 3]. Now, immune checkpoint inhibitors (CPIs) in the metastatic setting are associated with often durable response rates approaching 70% and three-year overall survival (OS) of up to 64% [4–6]. However, knowledge of predictors of response is lacking. Clinicians require a greater understanding of predictive markers for therapy selection, while researchers require an improved understanding of underlying mechanisms to inform drug development and trial design. Identification of molecular underpinnings of disease and clinical benefit from CPI is a promising avenue for addressing these unmet needs. Knepper and colleagues recently explored the correlation of clinical and molecular characteristics with benefit from immunotherapy in MCC [7]. In the present study, we build on this prior approach by incorporating multivariable analysis techniques, studying patients over a longer follow-up time period, and by studying a homogeneous MCC population all treated with CPIs. Additionally, we set out to include a greater number of CPI-treated patients in our work and statistically analyze the correlations of specific single nucleotide variants (SNVs) and copy number variations (CNVs) with response to CPIs.

## RESULTS

### Clinical characteristics

The cohort was comprised of approximately two thirds men and one third women with a median age of 71 (Table 1). Seven (16%) were immunosuppressed, including three patients with hematological malignances (acute myeloid leukemia, smoldering multiple myeloma, and chronic myeloid leukemia), two patients with autoimmune disease (with history of anti-tumor necrosis factor treatment but no active treatment during CPI), one with history of kidney transplant (receiving everolimus and prednisone), and one with recently diagnosed, active tuberculosis receiving rifamycin/isoniazid/pyrazinamide/ethambutol treatment. Primary site of disease was most commonly the skin of the limbs (63%) and head and neck (33%), and less frequently involved the trunk (7%). For eight patients (18%), the primary site of disease was unknown. The majority of the patients had pathologic stage III disease at diagnosis (62%, including 11 IIIA and 17 IIIB) (Supplementary Table 1). The majority of cases were either cN0 (12, 27%) or cN1 (20, 44%) at diagnosis. Pathologic nodal staging was similar: pN0 (11, 24%) and pN1 (20, 44%). Five patients (11%) had distant metastatic disease at diagnosis. Surgery with adjuvant radiation was the most common initial therapy for primary disease (44%), while 36% of patients received a chemotherapy-containing regimen as part of initial therapy.

**Table 1:** Demographics, clinical, and survival characteristics of patients with Merkel cell carcinoma treated with an immune checkpoint inhibitor (univariate analyses)

Characteristic	All (%)^A^, *N* = 45	Response^B^ (%), *N* = 17	No response (%), *N* = 22	*p*-value
**Median age in years (range)**	71 (39–89)	71 (50–89)	71 (50–89)	0.90
**Sex**				
Male	31 (69)	12 (71)	14 (64)	0.27
Female	14 (31)	5 (29)	8 (36)	
**Immune suppression**^C^				
Yes	7 (16)	2 (12)	4 (18)	0.29
No	38 (84)	15 (88)	18 (82)	
**Primary site of disease**				
Limb	19 (42)	8 (47)	10 (45)	-
Head and neck	15 (33)	5 (29)	8 (36)	
Unknown	8 (18)	3 (18)	4 (18)	
Trunk	3 (7)	1 (6)	0 (0)	
**Initial staging at diagnosis^D^**				
Stage I	9 (20)	5 (29)	2 (9)	-
Stage II	3 (7)	0 (0)	3 (14)	
Stage III	28 (62)	11 (65)	14 (64)	
Stage IV	5 (11)	1 (6)	3 (14)	
**Initial treatment regimen**				
Surgery and RT	20 (44)	7 (41)	10 (45)	0.435^D^
ChemoRT	10 (22)	4 (24)	4 (18)	
RT alone	6 (13)	2 (12)	4 (18)	
Surgery + ChemoRT	3 (7)	1 (6)	2 (9)	
Surgery alone	2 (4)	2 (12)	0 (0)	
Chemotherapy alone	2 (4)	0 (0)	1 (5)	
Surgery + Chemotherapy	1 (2)	1 (6)	0 (0)	
None	1 (2)	0 (0)	1 (5)	
**Number experiencing recurrence**	36 (80)	13 (76)	18 (82)	0.19
**Medianime to recurrence (months)**	4.8 (0.6–21.1)	3.7 (0.6–12.2)	6.3 (0.9–21.1)	0.10
**Initial staging at diagnosis of R/M disease^E^**				
Stage III	13 (29)	7 (41)	6 (27)	-
Stage IV	32 (71)	10 (59)	16 (73)	
**CPI line of therapy**				
Adjuvant	2 (4)	2 (12)	0 (0)	-
1	25 (56)	11 (65)	13 (59)	
2	13 (31)	3 (18)	7 (32)	
3	2 (4)	0 (0)	1 (5)	
4	2 (4)	1 (6)	1 (5)	
**CPI therapy**				
PD-1 inhibitor monotherapy	23 (51)	9 (53)	10 (45)	0.22 ^F^
PD-L1 inhibitor monotherapy	21 (47)	7 (41)	12 (55)	
PD-1/CTLA-4 dual therapy	1 (2)	1 (6)	0 (0)	
**Median baseline ALC (**× **10^6^/L) (range)**	780 (0–16,660)	680 (390–16,660)	875 (310–2,030)	0.30
**Immune-related adverse events^G^**				
Grade 2 or lower	37 (82)	12 (71)	20 (91)	0.09
Grade 3 or above	8 (18)	5 (29)	2 (9)	

Student’s *t*-test for continuous variables (age, number experiencing recurrence, and median baseline ALC), Fisher exact test for categorical variables (all other variables). All tests were univariate and two-sided. ^A^Except for continuous variables (age, time to recurrence, baseline absolute lymphocyte count); ^B^Response evaluation criteria in solid tumors, RECIST v1.1, 39 patients evaluable for radiographic response; ^C^Includes three patients with hematologic malignancy, two patients on treatment for autoimmune disease, one with history of kidney transplant, and one with active tuberculosis; ^D^Radiotherapy versus no radiotherapy ^E^American Joint Committee on Cancer (AJCC) 8th edition staging; ^F^PD-1 *vs.* PD-L1; ^G^Using Common Terminology Criteria for Adverse Events (CTCAE) version 5.0. ALC = absolute lymphocyte count, ChemoRT = concurrent chemoradiation, CPI = immune checkpoint inhibitor, R/M = recurrent/metastatic, RT = radiotherapy.

Thirty-six (80%) patients experienced disease recurrence, which occurred at a median of 4.8 months (range: 0.6–21) following the end of definitive therapy. The majority of patients were stage IV at the time of recurrence or metastasis (71%), and the remaining were stage III (29%). Nearly all patients received either anti-programmed cell death-1 (PD-1) monotherapy with pembrolizumab or nivolumab (23, 51%) or anti-programmed death-ligand 1 (PD-L1) monotherapy with avelumab (21, 47%), while one patient received combined anti-PD-1/anti-cytotoxic T-lymphocyte-associated protein 4 (CTLA-4) therapy. Most patients received CPI as first-line therapy in the metastatic setting (60%). Median absolute lymphocyte count (ALC) prior to administration of CPI was 780 × 10^6^ cells/liter (650 for patients who had received chemotherapy as part of initial therapy versus 970 for those who had not, *p* = 0.33). Eight (18%) patients experienced grade 3 or above immune-related adverse events (irAEs). These were autoimmune disorders of the respiratory system, gastrointestinal system, thyroid gland, joints, meninges, liver, adrenal gland, and heart.

### Response and survival outcomes

With a median follow-up time of 25.2 months, duration of therapy ranged from zero (one dose) to 26.3 months. Nine patients were still on therapy at the time of data collection. Among 39 evaluable patients (six patients did not undergo restaging scans following CPI initiation), confirmed objective responses to CPI were achieved in 17/39 (43%) patients (Supplementary Table 2). Of these, thirteen (33%) experienced a complete response (CR) and four (10%) experienced a partial response (PR). The median time to response was 2.1 months (range 0.1–12.0). Median duration of response was 24.2 months (95% confidence interval [CI] 8.8-not reached [NR]), with 86.6% of responders experiencing an ongoing response at six months and 70.0% of responders with an ongoing response at twelve months. Among the non-responders (56%), five (13%) experienced stable disease (SD) and seventeen (44%) progressive disease (PD). Response rate was numerically lower (but not to the degree of statistical significance) among patients who had distant metastatic disease at initial diagnosis compared to those initially diagnosed with local or locoregional disease (25% vs. 45%, *p* = 0.62). Having received any prior cytotoxic chemotherapy, whether in the curative or palliative setting, did not impact response rate (42% for chemotherapy-naïve vs. 46% chemotherapy-expereinced, *p* = 1.00). Response rate did not vary by specific CPI agent (47% for PD-1 inhibition, 37% for PD-L1 inhibition, and 0% for the single patient on dual PD-1/CTLA-4 inhibition, *p* = 0.23 by Fisher’s exact test for PD-1 vs. PD-L1). The clinical benefit rate (CBR), which combines the CRs, PR and SD rates, was 56%. Two patients received treatment through initial progression. Of these, one never experienced a response, while the other went on to experience a sustained CR starting two months after observed PD and lasting for over two years. Of the patients who experienced SD as best response, two were still on therapy at the time of data collection (one for over a year). The duration of treatment for the other three patients who experienced SD as best response were 4.2 months, 15.4 months, and 22.2 months.

**Figure 1:**
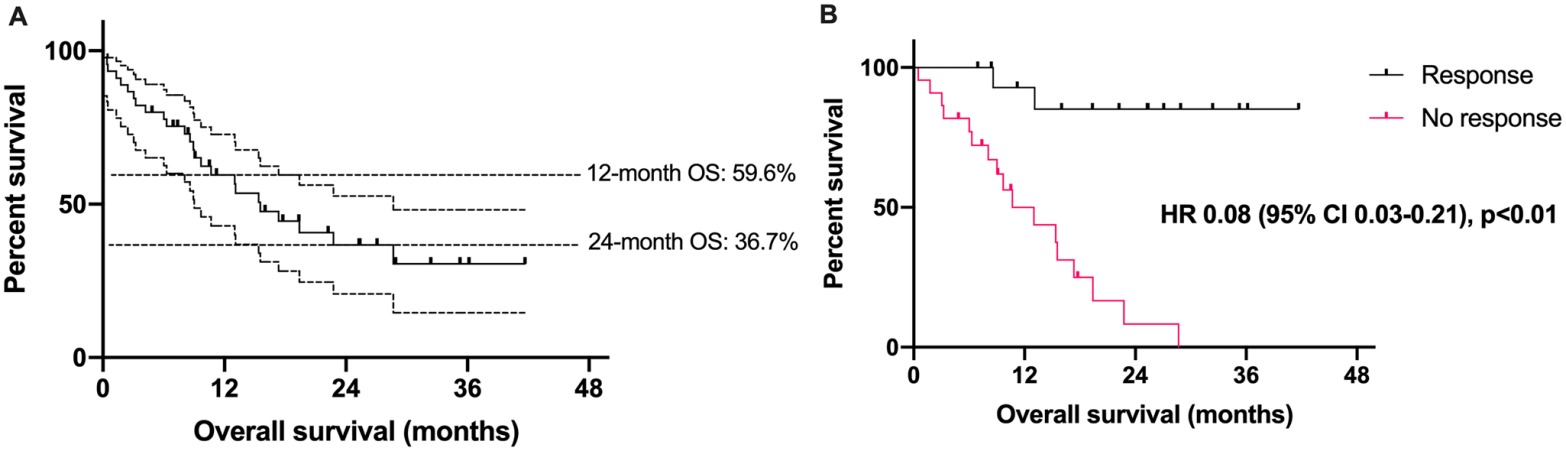
Survival outcomes in patients with Merkel cell carcinoma treated with an immune checkpoint inhibitor. (**A**) Overall survival (in months) among 45 patients with Merkel cell carcinoma treated with an immune checkpoint inhibitor. Dotted lines represent 95% confidence intervals. (**B**) Overall survival among patients with Merkel cell carcinoma based on response to immune checkpoint inhibitor. CI = confidence interval, HR = hazard ratio.

Median OS for the entire cohort was 15.5 months (95% CI 9.0–28.7). Six-month OS was 80.0% (95% CI 65.0–89.0), 1-year OS was 59.6% (95% CI 43.0–72.7), and 2-year OS was 36.7% (95% CI 21.1–52.8) (Figure 1). Median OS was not reached among responders, while median OS was 13.0 months among non-responders (HR 0.08; 95% CI 0.03–0.21, *p* < 0.01). Estimated 6-month OS was 100% among responders and 81.8% among non-responders, and estimated 2-year OS was 85.3 vs. 8.2%, respectively.

### Clinical predictors of outcomes

On multivariable analysis, patients with a higher stage at initial diagnosis of primary disease were less likely to respond to CPI therapy (odds ratio [OR] 0.06, *p* = 0.04, Table 2). Patients with a longer interval between the completion of initial treatment and recurrence (disease-free interval, DFI) were less likely to respond to CPI therapy (OR 0.75, *p* = 0.05). All other clinical, pathological and genetic factors failed to independently impact CPI response. No clinical, pathological or genetic features independently predicted survival outcomes on CPI therapy (Table 3) at this cohort size and for this duration of follow-up.

**Table 2:** The effect of clinical, pathologic, and genetic features on CPI response in Merkel cell carcinoma patients (multivariate logistic regression modeling)

Variable	Total (*n* = 39)		
	OR	[95% CI]	*p*-value
Female sex	3.00	0.05–163.94	0.59
Older age at diagnosis	0.91	0.78–1.06	0.24
Higher stage at diagnosis	0.06	< 0.01–0.92	**0.04**
Smoking history (current or former)	0.71	0.03–14.63	0.83
Immunosuppressed	0.05	< 0.01–2.87	0.15
Longer time to recurrence	0.75	0.56–0.99	**0.05**
Greater CPI line of therapy	1.21	0.35–4.24	0.76
Higher absolute lymphocyte count	1.12	0.69–1.82	0.64
Grade 3 or greater adverse events^A^	12.39	0.67–229.27	0.09
Higher total mutational burden	0.94	0.83–1.07	0.35
MCCP	0.28	0.01–13.39	0.52

Female sex, smoking history, immunosuppressed, grade 3 or greater adverse events, and MCCP are binary categorical variables. Stage at diagnosis and line of therapy are ordinal categorical variables. All other variables are continuous variables. Bolded *p*-values represent those below 0.05. Only includes patients evaluable for radiographic response. CI = confidence interval; CPI = immune checkpoint inhibitor; MCCP = Merkel cell carcinoma polyomavirus positive; OR = Odds ratio (response vs. non-response). ^A^Using Common Terminology Criteria for Adverse Events (CTCAE) version 5.0.

**Table 3:** The effect of clinical, pathologic, and genetic features on overall survival in patients with Merkel cell carcinoma treated with immune checkpoint inhibition (multivariate Cox proportional hazard modeling)

Variable	Total (*n* = 45)		
	HR	[95% CI]	*p*-value
Female sex	2.38	0.16–34.61	0.53
Older age at diagnosis	1.04	0.97–1.13	0.28
Higher stage at diagnosis	2.88	0.69–12.08	0.15
Smoking history (current or former)	3.19	0.55–18.57	0.20
Immunosuppressed	7.49	0.59–95.51	0.12
Longer time to recurrence	1.12	0.97–1.30	0.13
Greater CPI line of therapy	1.09	0.45–2.61	0.85
Higher absolute lymphocyte count	0.39	0.06–2.53	0.33
Grade 3 or greater adverse events ^A^	0.05	<0.01–1.08	0.05
Higher total mutational burden	1.02	0.93–1.11	0.68
MCCP	1.65	0.18–15.28	0.66

Female sex, smoking history, immunosuppressed, grade 3 or greater adverse events, and MCCP are binary categorical variables. Stage at diagnosis and line of therapy are ordinal categorical variables. All other variables are continuous variables. CI = confidence interval; CPI = immune checkpoint inhibitor; HR = hazard ratio (response vs. non-response); MCCP = Merkel cell carcinoma polyomavirus positive. ^A^ Using Common Terminology Criteria for Adverse Events (CTCAE) version 5.0.

### Molecular analysis

**Figure 2:**
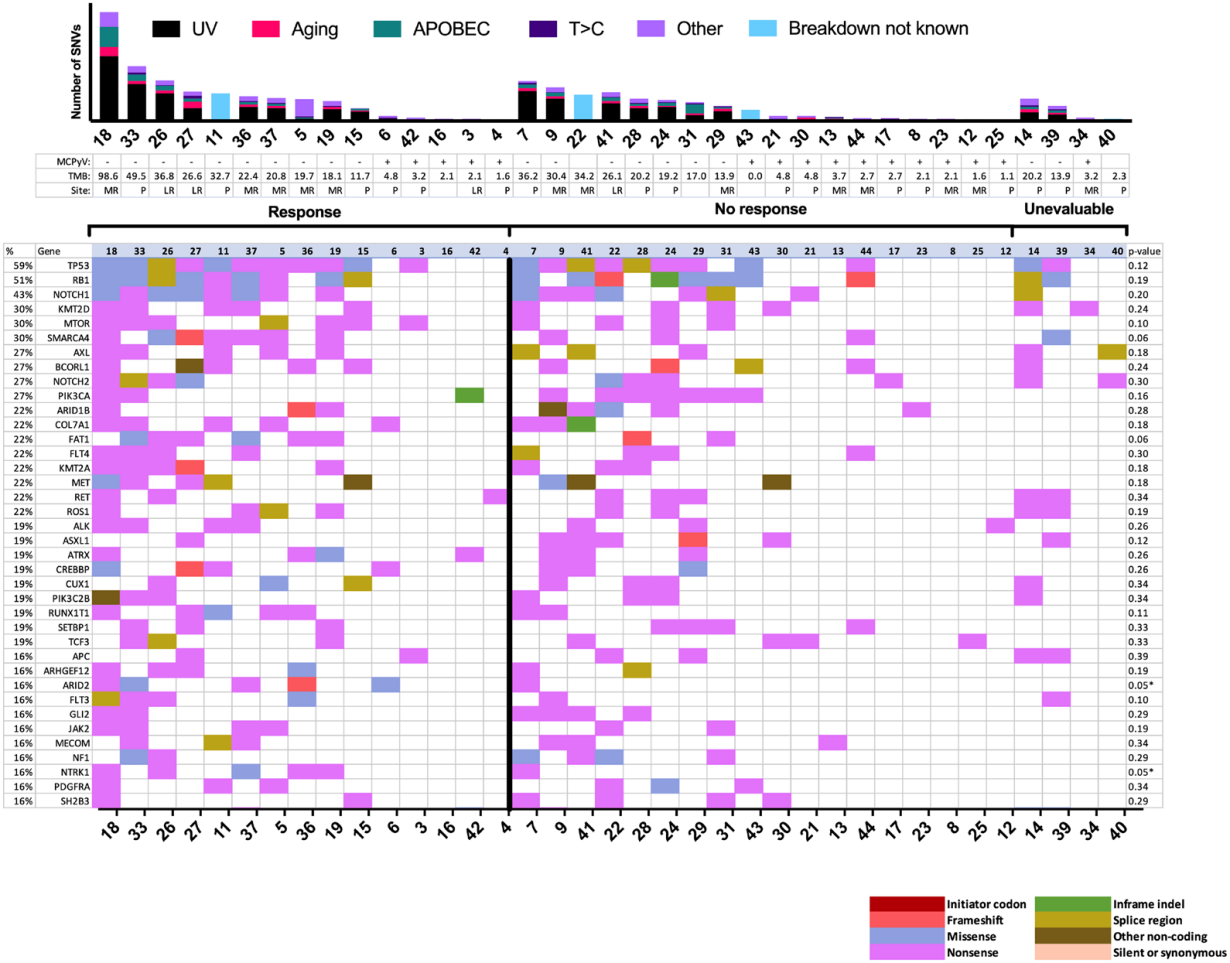
Mutational landscape by response to immune checkpoint inhibitor. Mutational plot showing the most frequently mutated genes (top-to-bottom, ≥15%) ordered by response and by total number of SNVs, with gene frequency listed at left (%), and Fisher exact test *p* values (response versus no response) at right. Asterisks denote values less than 0.05 (significant before Bonferroni correction, for which cutoff for significance is 0.0001 for our panel of 447 genes). The bar graph at top shows the total number of panel single nucleotide variants detected per sample by mutation signature. Blank MCPyV and TMB denote unknown values. LR = locoregional recurrence; MCPyV = Merkel cell polyomavirus status; MR = metastatic recurrence; P = primary site; SNV = single nucleotide variant; TMB = total mutational burden in mutations per Mb.

Of the 35/37 tumor samples whose Merkel cell polyomavirus (MCPyV) status could be inferred based on the genetic information from our panel, 16 (43%) were MCPyV-positive (MCCP) (Figure 2). Among responders, 5 (33%) were MCCP and 10 (67%) were MCPyV-negative (MCCN). Rates of MCPyV positivity were not significantly different among CPI responders and non-responders (*p* = 0.10 by univariable analysis) and did not correlate significantly with survival outcomes (Tables 2 and 3). Total mutational burden (TMB) was not significantly different among CPI responders and non-responders (median 19.7 versus 4.8 mutations per megabase, *p* = 0.11), and did not correlate significantly with survival outcomes. Rates of UV-related mutations did not correlate with outcomes.

**Figure 3:**
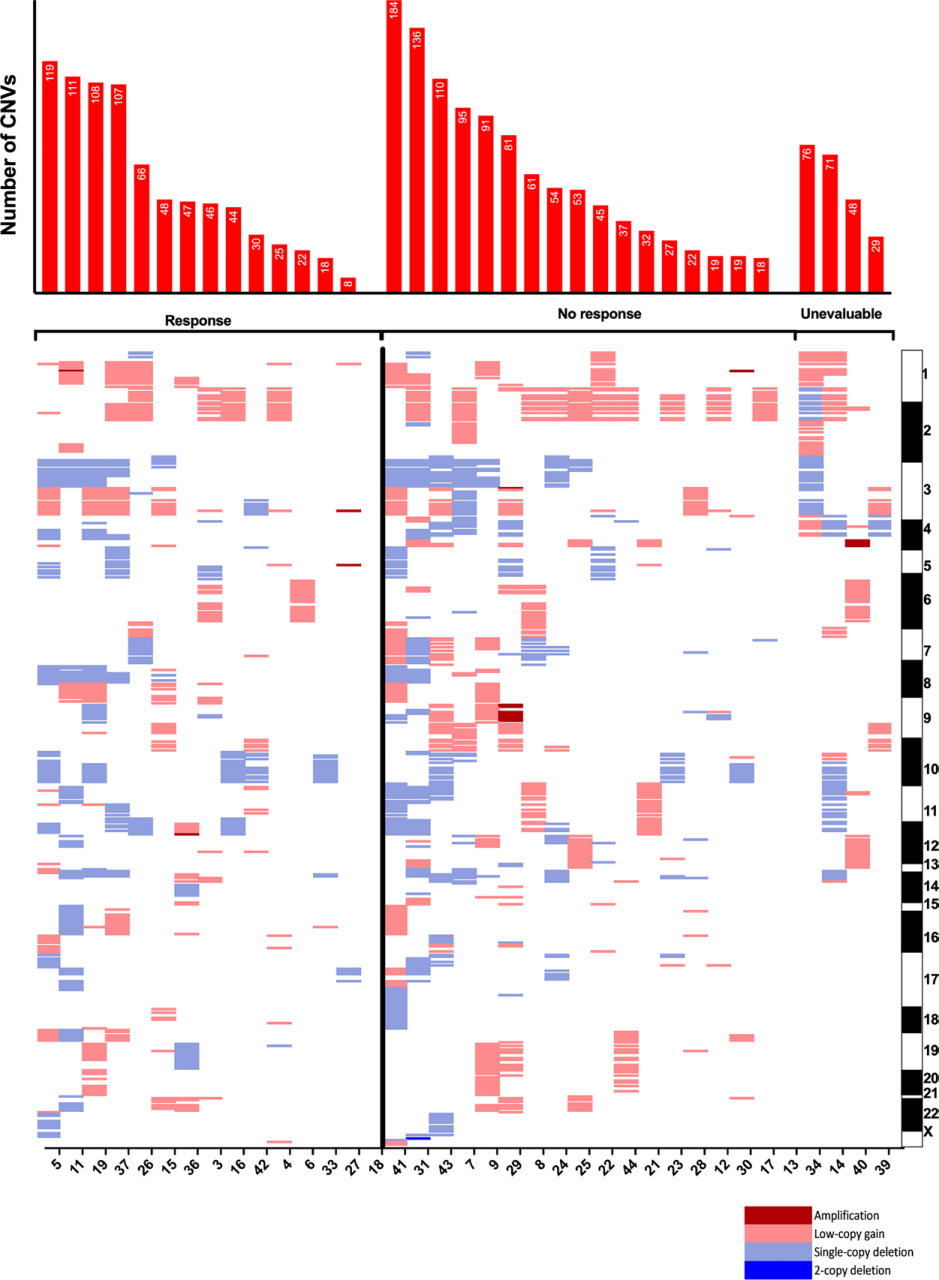
Copy-number variants by response to immune checkpoint inhibitor. Copy-number variants arranged by chromosomal band loci. Each column represents an individual tumor and corresponding gene loci. Chart at top shows total number of genes with copy number variants per sample. Patient samples presented by response and in order of descending total number of CNVs. CNV = copy-number variant.

The most common SNVs among the entire sequenced cohort were those in *TP53* (59%) and *RB1* (51%). Among responders, there were significantly more mutations in *ARID2* (*p* = 0.05) and *NTRK1* (*p* = 0.05) by traditional analysis, although these findings were not significant when considering the stricter Bonferroni cutoff for multiple testing (*p* < 1.1 × 10^-4^ required). *ARID2* and *NTRK1* SNVs were not significantly correlated with time to recurrence. Mutations in *SMARCA4* (*p* = 0.06) and *FAT1* (*p* = 0.06) trended towards being more frequent among responders. CNVs were analyzed among 33 patients (Figure 3). CNVs occurred at a median rate of 47 per sample (range: 0–184) and did not associate significantly with outcomes.

## DISCUSSION

Overall, our cohort of patients with CPI-treated MCC demonstrated the strong efficacy (60% 12-month OS and 37% 24-month OS) that clinicians have come to expect with immunotherapy in this setting. As anticipated, individuals who experienced a response to CPIs also experienced longer OS. The demographics of our cohort appears to largely match those of the U.S. MCC population: median age of 71 years in our cohort versus 75–79 in epidemiologic studies, roughly two-thirds men [1]. Our cohort deviates from expected prevalence of MCCP disease (43% in our cohort, versus roughly 80% expected across all individuals with MCC) [8]. This discrepancy may reflect the advanced, aggressive disease of patients in our cohort, as MCCP tumors tend to have better prognoses [9]. The disparity is also likely due, to some degree, to chance.

We observed a similar response rate to those recorded by Knepper and colleagues in their previous study that explored genomics and clinical benefit from CPIs (43% versus 44%) [7]. To our knowledge, there was no overlap between the patient sample in the Knepper study and our study. As the studies used different platforms for genomic testing, the genomic data between the two studies are distinct. As was the case in the previous study, we did not observe a correlation between either TMB or MCPyV status and clinical benefit from CPIs. Both studies identified *TP53* and *RB1* as the most commonly altered genes across each full cohort. Unlike the prior study, we included analysis of DFI, time to response, duration of response, survival time, and statistical tests related to the correlation of SNVs and CNVs with response.

After controlling for other clinical parameters, longer time to recurrence was associated with lower odds of response to CPI. We speculate this finding may be related to prior radiotherapy, as patients with node-positive disease are both more likely to recur sooner and are more likely to have received radiotherapy (and may thus be more likely to benefit from CPIs) [10]. Data in non-small cell lung cancer has indicated that patients with prior exposure to radiotherapy tend to have disease that is more likely to respond to immunotherapy [11]. The potential mechanisms through which immunotherapy and radiotherapy may synergize (e.g., radiotherapy-induced activation of immune cells and antigen presentation) have been widely studied and described [12–17]. Since only six patients in our study did not receive radiotherapy as part of initial treatment, we cannot make any definitive statements about radiotherapy’s association with CPI response. On multivariable analysis, patients with less advanced (earlier stage) disease at diagnosis were also more likely to experience a response to CPI. This observation reflects the impressive 71% CR rate to CPIs among radiographically evaluable patients who had originally been diagnosed with stage I disease, received definitive therapy, then experienced disease recurrence. Similarly, our previous work has demonstrated an association between lower total tumor burden and greater likelihood of response to CPIs in squamous cell carcinoma of the head and neck [18]. However, given that only a small number of patients in our cohort had stage I or stage II disease, and given the wide 95% CI for this covariable, we defer any conclusions related to this observation for the current study.

Response rates in our study did not vary significantly by presence of metastatic disease at diagnosis, by any prior chemotherapy exposure, or by line of therapy for advanced disease. These observations are largely in line with previous studies, which have demonstrated trends towards improved response in patients who have received fewer prior systemic agents, but not to a degree of statistical significance [4, 6, 19]. Such trends may reflect the rarity of this disease and no study being sufficiently powered to detect differences of this variety. The present cohort, for example, included only four radiographically-evaluable patients with metastatic disease at initial diagnosis, only one of whom experienced a radiographic response. The 25% response rate among those with metastatic disease at diagnosis indeed could, in larger studies, differ significantly from the 45% response rate among those with local or locoregional disease at diagnosis. Our study is limited by its retrospective nature and, given the rarity of the disease, small sample size.

The genomic landscape of the tumors in our trial were largely concordant with existing literature. Our study shared the top three most commonly mutated genes, *TP53, RB1,* and *NOTCH1,* with a recent retrospective study of a similar-sized cohort [7]. In line with this previous study, we observed a predominantly ultraviolet-associated mutational pattern in nearly all samples. Degree of ultraviolet signature predominance in a given sample did not associate with clinical benefit from CPIs. Also consistent with the prior work, TMB did not associate significantly with response, likely due to small sample size. SNVs in the *ARID2* and *NTRK1* genes correlated with CPI response, although this association was not significant after Bonferroni correction. All but one patient with *ARID2*-mutated tumors in our cohort experienced response to CPIs. The association between *ARID2* and immune function has previously been established in melanoma [20]. The ARID2 protein is involved in the regulation of chromatin remodeling and its loss of function is associated with increased tumor cell sensitivity to interferon-γ and expression of T-cell cytotoxicity genes. Previous work has identified *ARID2* as a commonly mutated gene in MCC [7].

*NTRK1* codes for tropomyosin receptor kinase A (TrkA), a tyrosine receptor kinase that feeds into the MAPK pathway. TrkA expression may be characteristic of MCC: In one MCC case series, all 36 specimens exhibited cytoplasmic TrkA, although staining was generally weak (only 2 (6%) samples stained strongly for TrkA). [21] Its ligand, nerve growth factor (NGF) is expressed by tumor-associated interdigitating cells in roughly 70% of MCC cases – a sensical observation for a tumor of neuroendocrine origin [22]. A small study (*n* = 10) revealed that MCCP tumors may tend to display activating alternative splicing of *NTRK1* mRNA. [23] Notably, none of the samples with *NTRK1* SNVs in our cohort were MCCP. If TrkA activity is a key oncogenic pathway for MCCP tumors, we could speculate that *NTRK1* alterations may confer an “MCCP-like” phenotype onto MCCN tumors by activating this pathway.

Specific protein changes in *NTRK1* may be particular to MCC, as none of the amino acid changes observed in our cohort (Supplementary Table 3) have been seen in The Cancer Genome Atlas program [24]. As such, it is difficult to determine the significance of the specific amino acid changes in our cohort. One specific amino acid change, D668N, has been studied previously and was shown to activate tyrosine kinase activity of TrkA [25]. Evidence from lung cancer models, in which *NTRK1* activity has been shown to inhibit the effect of CPIs via several mechanisms, including the promotion of T-cell exhaustion, offer some rational for some interaction between the two pathways [26]. In particular, repressing *NTRK1* signalling could enhance tumoral benefit from CPIs. Our finding may provide support for the study of Trk inhibitors such as larotrectinib in advanced MCC. Furthermore, *ARID2* and *NTRK1* alterations could serve as predictive markers, in the context of other clinical and pathologic findings, as clinicians make management decisions related to CPIs in patients with MCC. Our study is limited by not including gene expression analysis – future work that includes such methods can help elucidate underlying driving mechanisms.

Our study points to several factors for clinicians to consider in the context of other clinical and pathologic findings when making treatment decisions. In particular, patients with *ARID2* mutations, *NTRK1* mutations, or shorter time to recurrence may be expected to have a higher likelihood of benefit from CPIs. These findings present potential areas for future basic scientific research related to molecular mechanisms. Future clinical study may explore the potential for Trk inhibition in combination or sequence with immunotherapies.

## MATERIALS AND METHODS

### Study cohort

Forty-five patients with biopsy-proven MCC treated with an immune checkpoint inhibitor-containing regimen at the Dana-Farber Cancer Institute from 2013 to 2020 were retrospectively identified following expedited institutional review board approval. We obtained tumor tissue samples from 37 (82%) patients for molecular testing (other samples were either exhausted or not available). Among these, 17 (46%) samples came from a primary tumor site, four (11%) came from an area of locoregional recurrence, 13 (35%) were derived from a distant metastatic focus, and site classification was unknown for three (8%). Demographics, clinical, and survival characteristics, along with clinical and pathological features were collected from patient electronic health medical records. Response data was estimated from clinical radiology reports and physician notes based on Response Evaluation Criteria in Solid Tumors, RECIST v1.1 [27].

### Molecular analysis

All patients sequenced in this study were consented individually to the Dana-Farber institutional Cancer Research Study (protocols 11-104 and 17-000) and a clinical laboratory improvement amendments (CLIA)-certified laboratory performed molecular testing. The molecular testing was performed on tissue samples with at least 50% viable malignant cell quality, and with at least 20% tumor on hematoxylin and eosin slide review. As previously described, the most updated iteration of this molecular testing (version 3) analyzes tumor tissue for a panel of known cancer-related mutations within the protein-coding portions of 447 genes [28, 29]. TMB was calculated as the number of non-synonymous somatic mutations per megabase of exonic sequence data across all genes on the panel. Tumors were assessed for likely MCPyV status based on genetic information, including data gathered using ViroPanel, an expansion of our institutional OncoPanel platform that captures oncogenic viral sequences, where available [30, 31]. Mechanistic origin of single nucleotide and dinucleotide variants was determined as described previously [30]. Sequencing data is available from the authors upon request.

### Statistical analysis

Student *t*-test was used for continuous variables and a Fisher exact was used for categorical variables to compare clinical characteristics by response. The Kaplan-Meier method and log-rank testing were used for survival analyses. All tests were two-sided. All tests were univariate except for multivariate analysis of odds ratio of response and hazard ratio for death by response to immunotherapy. We compared the results of statistical tests with and without a Bonferroni correction, when appropriate. Our study had a 6% power to detect a 10% difference in odds ratio based on response. Data were analyzed using Stata/IC (version 16.1).

## SUPPLEMENTARY MATERIALS




