# Supplementary material for: Predictors of immunotherapy benefit in Merkel cell carcinoma
Source: Oncotarget. 2020 Nov 24;11(47):4401–10. doi: 10.18632/oncotarget.27823 (PMC7720777; doi:10.18632/oncotarget.27823)
Supplement: Supplementary file 1 [file oncotarget-11-4401-s001.pdf]

Predictors of immunotherapy benefit in Merkel cell carcinoma

SUPPLEMENTARY MATERIALS

Supplementary Table 1: Details of staging at diagnosis

	Number of patients
Stage IIIA	11
Stage IIIB	17
cN0	12
cN1	20
cN2	5
cN3	7
cNX	1
pN0	11
pN1	20
pN2	5
pN3	7
pNX	2

"c" indicates clinical staging, "*p*" indicates pathologic staging.

Supplementary Table 2: Radiographic best response to immune checkpoint inhibitor therapy

Best response	Total (*n* = 39)
	Number (%)
Complete response	13 (33)
Partial response	4 (10)
Stable disease	5 (13)
Progressive disease	17 (44)

Table includes the 39 patients with evaluable disease (underwent restaging scans following immune checkpoint inhibitor initiation).

Supplementary Table 3: Specific amino acid alterations at single nucleotide variant sites. See Supplementary Table 3
