# Supplementary material for: Predictors of immunotherapy benefit in Merkel cell carcinoma
Source: Oncotarget. 2020 Nov 24;11(47):4401–10. doi: 10.18632/oncotarget.27823 (PMC7720777; doi:10.18632/oncotarget.27823)
Supplement: Supplementary file 2 [file oncotarget-11-4401-s002.docx]

**Supplementary Table 3: Specific amino acid alterations at single nucleotide variant sites**

| Gene | **18** | **33** | **26** | **27** | **11** | **37** | **5** | **36** | **19** | **15** | **6** | **3** | **16** | **42** | **4** | **7** | **9** | **41** | **22** | **28** | **24** | **29** | **31** | **43** | **30** | **21** | **13** | **44** | **17** | **23** | **8** | **25** | **12** | **14** | **39** | **34** | **40** |
| --- | --- | --- | --- | --- | --- | --- | --- | --- | --- | --- | --- | --- | --- | --- | --- | --- | --- | --- | --- | --- | --- | --- | --- | --- | --- | --- | --- | --- | --- | --- | --- | --- | --- | --- | --- | --- | --- |
| TP53 | p.R196* | p.Q38* |  | p.R248W |  | p.E258K | p.I195F | p.V157F | p.E258K | p.R342* |  | p.S215R |  |  |  | p.R196* | p.G266R |  | p.V218G |  | p.E285K | p.G187S |  |  |  |  |  | p.C135F |  |  |  |  |  | p.R213* | p.C135F |  |  |
| RB1 | p.R579* | p.W99* |  | p.R787* |  | p.W78* | p.S560F |  | p.R556* | p.V654_splice |  |  |  |  |  | p.Q383* |  |  | p.N123Tfs*2 |  | p.459_463SMLKS>* | p.R787* | p.W516* |  |  |  |  | p.R876Pfs*3 |  |  |  |  |  |  | p.Q62* |  |  |
| NOTCH1 | p.E1024* | p.P380L | p.Q58* | p.Q462* |  | p.Q1957* | p.G347N |  | p.G546E |  |  |  |  |  |  | p.Q1247* | p.P2222L |  | p.W1813* |  |  | p.P224S |  |  |  | p.H2500D |  |  |  |  |  |  |  |  |  |  |  |
| KMT2D | p.S2590W | p.P1945L |  |  |  | p.P2190S |  |  |  | p.T1986I |  |  |  |  |  | p.P1232S |  |  |  |  | p.S2969L |  | p.T5466I |  | p.T5067M |  |  |  |  |  |  |  |  | p.D1734N |  | p.D1378H |  |
| MTOR | p.T1723I | p.P2308S | p.L268F |  |  |  |  |  | p.P116S | p.M1174I |  | p.A469T |  |  |  | p.P765F |  |  | p.S2155F |  | p.P1408L |  | p.H966Y |  |  |  |  |  |  |  |  |  |  |  |  |  |  |
| SMARCA4 | p.P304S |  | p.R1093* | p.Q194Sfs*109 |  | p.G1162S | p.P18L |  | p.V1228M |  |  |  |  |  |  |  | p.R1613W |  |  |  | p.L1222F |  |  |  |  |  |  | p.R1192C |  |  |  |  |  |  | p.Q1606* |  |  |
| AXL | p.S285L | p.L471F |  |  |  |  | p.R533W |  | p.R507W |  |  |  |  |  |  |  |  |  |  |  |  | p.A457G |  |  |  |  |  |  |  |  |  |  |  | p.E818K |  |  |  |
| BCORL1 | p.S659F |  |  |  |  |  |  | p.R789P |  | p.P184S |  |  |  |  |  |  | p.P1004L |  |  |  | p.I405fs |  |  |  |  |  |  | p.P190T |  |  |  |  |  | p.P600S |  |  |  |
| NOTCH2 | p.P1371S |  | p.G1386K | p.R1933* |  |  |  |  |  |  |  |  |  |  |  |  |  |  | p.Q1086* | p.D1561N | p.S2136R |  |  |  |  |  |  |  | p.R1563H |  |  |  |  | p.V1064L |  |  |  |
| PIK3CA | p.E545K | p.L866W |  |  |  |  |  |  |  |  |  |  |  | p.H450delinsGGKKN |  |  | p.H1047L |  | p.E81K | p.E542K | p.L339F | p.E542K | p.G414R |  |  |  |  |  |  |  |  |  |  |  |  |  |  |
| ARID1B | p.R1670T |  |  |  |  |  |  | p.Y926Tfs*8 | p.V2001F |  |  |  |  |  |  |  |  |  | p.Q54* |  | p.P749S |  |  |  |  |  |  |  |  | p.G293A |  |  |  |  |  |  |  |
| COL7A1 | p.R293Q | p.G2466K |  |  |  |  | p.P644L |  |  |  | p.P867L |  |  |  |  | p.G2614K | p.Q1460K |  |  |  |  |  |  |  |  |  |  |  |  |  |  |  |  |  |  |  |  |
| FAT1 |  | p.Q3192* | p.R1099C | p.T1563I |  | p.R2597* |  | p.G4275V | p.E2500K |  |  |  |  |  |  |  |  |  |  | p.G19Wfs*4 |  |  | p.D2630N |  |  |  |  |  |  |  |  |  |  |  |  |  |  |
| FLT4 | p.E1180K | p.G603R | p.S1249F |  |  | p.S537F |  |  |  |  |  |  |  |  |  |  |  |  |  | p.V1090M | p.G1317E |  |  |  |  |  |  | p.E646K |  |  |  |  |  |  |  |  |  |
| KMT2A | p.L3459I | p.E1468K | p.S982Y | p.R2622Lfs*16 |  |  |  |  | p.R1350H |  |  |  |  |  |  | p.P1354S |  |  | p.P1705L | p.S3517F |  |  |  |  |  |  |  |  |  |  |  |  |  |  |  |  |  |
| MET | p.G1085* | p.N45D |  | p.E28K |  |  |  |  |  |  |  |  |  |  |  |  | p.R731* |  |  |  |  |  |  |  |  |  |  |  |  |  |  |  |  |  |  |  |  |
| RET | p.G453R |  | p.G792R |  |  |  |  |  |  |  |  |  |  |  | p.V648I |  |  |  | p.R99W |  | p.T451M | p.S705F |  |  |  |  |  |  |  |  |  |  |  | p.E107K | p.R180Q |  |  |
| ROS1 | p.P1539L |  |  |  |  | p.N2273S |  |  | p.S1664F |  |  |  |  |  |  |  |  |  | p.L2133S |  | p.R863P |  |  |  |  |  |  |  |  |  |  |  |  | p.E1257K | p.G697R |  |  |
| ALK | p.G816E | p.G902E |  |  |  | p.P336S |  |  |  |  |  |  |  |  |  |  |  |  |  |  |  | p.G1137R |  |  |  |  |  |  |  |  |  |  | p.N1394H |  |  |  |  |
| ASXL1 |  |  |  | p.S1281F |  |  |  |  |  |  |  |  |  |  |  |  | p.P1330F |  | p.H315Y |  |  | p.W960Dfs*9 |  |  | p.S1391R |  |  |  |  |  |  |  |  |  | p.S1223F |  |  |
| ATRX | p.E410K |  |  |  |  |  |  | p.R1687G | p.R418* |  |  |  |  | p.K445N |  |  | p.L1981I |  |  |  |  | p.D1222N |  |  |  |  |  |  |  |  |  |  |  |  |  |  |  |
| CREBBP | p.Q503* |  |  | p.I1084Sfs*15 |  |  |  |  |  |  | p.L6P |  |  |  |  |  | p.T2073I |  |  |  |  | p.S1263* |  |  |  |  |  |  |  |  |  |  |  |  |  |  |  |
| CUX1 |  |  | p.M184I |  |  |  | p.W879* |  |  |  |  |  |  |  |  |  | p.L1459V |  |  | p.E128D | p.P1247L |  |  |  |  |  |  |  |  |  |  |  |  | p.A17T |  |  |  |
| PIK3C2B |  | p.P173L | p.D687N |  |  |  |  |  |  |  |  |  |  |  |  | p.S535F |  |  |  | p.S852L | p.P1478L |  |  |  |  |  |  |  |  |  |  |  |  | p.E547K |  |  |  |
| RUNX1T1 | p.R31C |  |  | p.A90T |  |  | p.E444K | p.S397I |  |  |  |  |  |  |  | p.R31C | p.D347N |  |  |  |  |  |  |  |  |  |  |  |  |  |  |  |  |  |  |  |  |
| SETBP1 |  | p.P759L |  | p.S1587F |  |  |  |  | p.K1341E |  |  |  |  |  |  |  |  |  |  |  | p.R401Q | p.L951F | p.P906L |  |  |  |  | p.T1010I |  |  |  |  |  |  |  |  |  |
| TCF3 |  | p.G376D |  |  |  |  |  |  | p.R158Q |  |  |  |  |  |  |  |  |  |  |  |  |  |  |  | p.A8V | p.D480N |  |  |  |  |  | p.G99R |  |  |  |  |  |
| APC |  |  |  | p.A2046T |  |  |  |  |  |  |  | p.H1349Q |  |  |  |  |  |  | p.L93V |  |  | p.S1389F |  |  |  |  |  |  |  |  |  |  |  | p.S1559F | p.R230C |  |  |
| ARHGEF12 | p.P1393L |  | p.D914N | p.R71C |  |  |  | p.G107* |  |  |  |  |  |  |  | p.S138F |  |  |  |  |  |  |  |  |  |  |  |  |  |  |  |  |  |  |  |  |  |
| ARID2 | p.P1497S | p.W1669* |  |  |  | p.K235N |  | p.G1291Efs*4 |  |  | p.R1297* |  |  |  |  | p.S130F |  |  |  |  |  |  |  |  |  |  |  |  |  |  |  |  |  |  |  |  |  |
| FLT3 |  | p.E858K | p.G757K |  |  |  |  | p.E346* |  |  |  |  |  |  |  |  | p.G697D |  |  |  |  |  |  |  |  |  |  |  |  |  |  |  |  |  | p.G613E |  |  |
| GLI2 | p.E500K | p.S79F |  |  |  |  |  |  |  |  |  |  |  |  |  | p.A1576V | p.S1542F |  |  | p.S863L |  |  |  |  |  |  |  |  |  |  |  |  |  |  |  |  |  |
| JAK2 | p.R761K | p.K1053R |  |  |  | p.D789N | p.L1119Q |  |  |  |  |  |  |  |  |  |  |  | p.P58S |  |  |  | p.R487C |  |  |  |  |  |  |  |  |  |  |  |  |  |  |
| MECOM |  | p.P574S |  |  |  | p.P523S |  |  |  |  |  |  |  |  |  |  | p.P316S |  |  |  |  |  |  |  |  |  | p.P276L |  |  |  |  |  |  |  |  |  |  |
| NF1 |  | p.E806* | p.R2349H |  |  |  |  |  |  |  |  |  |  |  |  | p.Q756* |  |  | p.W1831* |  |  |  | p.S285F |  |  |  |  |  |  |  |  |  |  |  |  |  |  |
| NTRK1 | p.D668N |  | p.E413K |  |  | p.Q769* |  | p.K100N | p.S465F |  |  |  |  |  |  | p.G661K |  |  |  |  |  |  |  |  |  |  |  |  |  |  |  |  |  |  |  |  |  |
| PDGFRA | p.E289K |  |  |  |  |  | p.L507F |  |  |  |  |  |  |  |  |  |  |  | p.F837L |  | p.W549* |  |  |  |  |  |  |  |  |  |  |  |  |  |  |  |  |
| SH2B3 | p.A42V |  |  |  |  |  |  |  |  | p.G409E |  |  |  |  |  | p.A22V |  |  | p.P155L |  |  |  | p.P85L |  | p.D356E |  |  |  |  |  |  |  |  |  |  |  |  |
